# Distribution and Risk of Mycolactone-Producing Mycobacteria Transmission within Buruli Ulcer Endemic Communities in Côte d’Ivoire

**DOI:** 10.3390/tropicalmed2010003

**Published:** 2017-02-26

**Authors:** Christelle Dassi, Lydia Mosi, Charles A. Narh, Charles Quaye, Danièle O. Konan, Joseph A. Djaman, Bassirou Bonfoh

**Affiliations:** 1 UFR Biosciences, Université Félix Houphouët Boigny, Abidjan, 01 BP V 34 Abidjan 01, Ivory Coast; christelledassi@gmail.com (C.D.); djamanj@yahoo.fr (J.A.D.); 2 Department of Environment and Health, Centre Suisse de Recherches Scientifiques en Côte d’Ivoire, Adiopodoumé, 01 BP 1303, Abidjan 01, Ivory Coast; CakugbeyNarh@noguchi.ug.edu.gh (C.A.N.); CQuaye@noguchi.ug.edu.gh (C.Q.); konandaniele@yahoo.fr (D.O.K.); bassirou.bonfoh@csrs.ci (B.B.); 3 Biochemistry, Cell and Molecular Biology Department, University of Ghana, Legon, P. O. Box LG 54, Legon, Accra, Ghana; 4 West African Centre for Cell Biology of Infectious Pathogens, University of Ghana, Legon, P. O. Box LG 54, Legon, Accra, Ghana; 5 Parasitology Department, Noguchi Memorial Institute for Medical Research, Legon, P. O. Box LG 581, Legon, Accra, Ghana

**Keywords:** mycolactone-producing mycobacteria, environment, human, transmission, Buruli ulcer, VNTR typing, phylogenetics

## Abstract

In Buruli ulcer (BU) endemic communities, most mycolactone-producing mycobacteria (MPM), including *Mycobacterium ulcerans*, the causative agent, are present in water bodies used by inhabitants; yet, their mode of transmission is still unclear. This study aimed to assess the distribution of MPM strains, both from human suspected cases and aquatic environments, for identifying possible transmission modes within two BU endemic districts, Daloa and Tiassalé (Taabo), in Côte d’Ivoire. Collected samples were processed using conventional polymerase chain reaction and screened for the presence of non-tuberculous mycobacteria (NTM) and MPMs using 16S rRNA, IS*2404* and enoyl reductase (ER) primers. MPM-positive samples were further discriminated using variable number tandem repeat (VNTR) typing and sequencing. 16S rRNA and IS*2404* sequences confirmed that 94% of the clinical samples contained MPMs. For environmental samples, 53% were contaminated with NTMs, of which 17% contained MPMs particularly *M. ulcerans*, suggesting that water-related activities could predispose inhabitants to BU transmission. MPM discrimination by VNTR at four *M. ulcerans* Agy99 loci identified genotype C, previously reported in Côte d’Ivoire as the most dominant profile. Phylogenetic clustering on the basis of genetic diversity in the MIRU 1 locus showed two main *M. ulcerans* lineages in Côte d’Ivoire.

## 1. Introduction

Buruli ulcer (BU) is a necrotizing skin disease that has a huge socio-economic impact on affected individuals, their families and the community at large [1]. It is the third most common mycobacterial disease after tuberculosis and leprosy [2] and is endemic in more than thirty countries, particularly West Africa, including rural parts of Cameroon, Togo, Benin, Ghana and Côte d’Ivoire. About 48% of reported cases in Africa are in children below 15 years, and both genders are equally affected.

The causative microorganism, *Mycobacterium ulcerans*, is an environmental non-tuberculous mycobacterium (NTM), which produces mycolactone as its main virulence factor [1]. Recently, other mycolactone-producing mycobacteria (MPM) have been identified as novel species causing disease in fishes and frogs [3,4]. MPMs are therefore of huge importance to public health due to the devastating diseases they cause in humans and animals.

The transmission of these mycobacteria is still poorly understood, making it difficult to implement effective prevention and control strategies. Public health control efforts have focused on sensitization, active case detection and early treatment of infections in endemic communities [1,5,6,7]. Socio-economic activities around wetlands or slow-moving water bodies have been suggested as risk factors of infection [8,9,10,11]. Because of the difficulty in culturing *M. ulcerans* from aquatic environments [12], environmental studies have focused on the detection of *M. ulcerans* DNA [13,14,15,16]. These advances have enhanced our understanding of the epidemiology of some MPMs and improved the diagnosis of *M. ulcerans*.

PCR detection of mycobacterial DNA using 16S rRNA, the insertion sequences (IS*2404* and IS*2606*) and genes encoding the enoyl and keto reductase (ER/KR) enzymes involved in the synthesis of *M. ulcerans* mycolactone have increased the rate of mycobacterium identification in clinical and environmental samples [13,15,17]. Additional discriminative tools including variable number tandem repeat (VNTR) and single-nucleotide polymorphism (SNP) typing have also been developed and successfully used to differentiate between MPMs [18,19,20,21,22,23,24].

Using these tools, we previously showed that individuals in BU endemic communities in Ghana may be infected from *M. ulcerans*-contaminated water bodies with which they frequently come into contact [16]. Thus, using similar approaches, we sought (1) to understand the distribution of MPMs in five BU endemic communities in Côte d’Ivoire, (2) to compare MPM strains, particularly *M. ulcerans* genotypes, between patients and environmental samples in water bodies they come into contact with, and (3) to determine the behavioral activities that might predispose inhabitants to *M. ulcerans* infection.

## 2. Materials and Methods

### 2.1. Ethical Approval and Questionnaire Administration

The study was conducted in accordance with the Declaration of Helsinki and received approval from the Ethics and Research National Committee (CNER) of the Health and AIDS control Ministry of Côte d’Ivoire (No. 3320/MSLS/CNER-P; Ministère de la Santé et de la Lutte contre le SIDA/Comité National d’Ethique et de la Recherche/Président). This was a cross-sectional study where the participants from the study communities were interviewed on their perception of BU disease. Samples were collected from patients who consented during an active case detection. The two activities were conducted at different time points with interviews preceding surveillance. The study was explained to participants, and informed written consent for each patient, or parent in the case of children, was obtained before enrolment into the study. Participants with confirmed BU infection, but not on treatment, were referred to a nearby approved BU clinic for treatment.

### 2.2. Study Communities

Study sites were selected based on BU endemicity data provided by the National Program for BU control (PNLUB). Selected communities were located in two major BU endemic areas in Côte d’Ivoire [25] with proximal and high-use aquatic ecosystems, including slow-moving water bodies. Daloa is a district (Haut-Sassandra) in the western centre approximately 406 km from the economic capital, Abidjan. This area is largely covered with forest and has the Sassandra River tributaries, which are used for irrigation, fishing and domestic activities. Gorodi and Zaïbo were the two selected rural communities from Daloa. Three additional communities, Sokrogbo, Léléblé and Ahondo, within Taabo area (Tiassalé district) were also included in this study. Taabo is in the southeastern part of the country approximately 160 km from Abidjan. Located within Taabo is a hydroelectric dam built over the Bandama River. There are several displaced villages along the river with associated socio-economic activities. Social and demographic characteristics of the population, as well as the frequency of contact with various aquatic environments were estimated by the use of structured questionnaires.

### 2.3. Administration of Questionnaire

As part of the bigger study, five hundred questionnaires were administered in the study communities from 12 February to 4 March 2012. The questionnaires mainly elicited information on the demographic characteristics of the community inhabitants, the main occupations of households, contact rates of the community members with various aquatic environments and animals, common diseases, BU knowledge and perception, and prevention and treatment. Communities were stratified into subsets based on population and reported cases of BU. Questionnaires were mainly administered to members of the household in the French language. Local languages were used only to verify unclear responses using the help of a trained local community aid.

### 2.4. Collection of Human Samples

**Figure 1:**
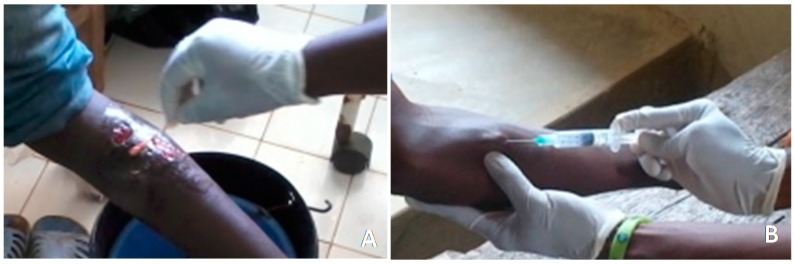
Sample collection from suspected cases. (**A**) Swab specimen collected from ulcer on a patient’s leg; (**B**) fine needle aspiration collected from a nodule on a patient’s arm.

Besides the administration of questionnaires, active case surveillance of BU suspected cases was undertaken with support from trained staff in the community health centers. Sample collection was performed during the dry season (17 to 26 September 2012) after questionnaire administration in the communities on both treated and untreated patients within the communities using protocols previously described [16]. Briefly, fine needle aspirations (FNA) or multiple swabs were collected at the health center by the medical staff in each community, depending on the type of lesion (nodule, edema or ulcer) (Figure 1). Each collected sample was placed in a sterile sample tube, which was labelled and stored on ice for transport to the laboratory.

### 2.5. Environmental Sampling

**Figure 2:**
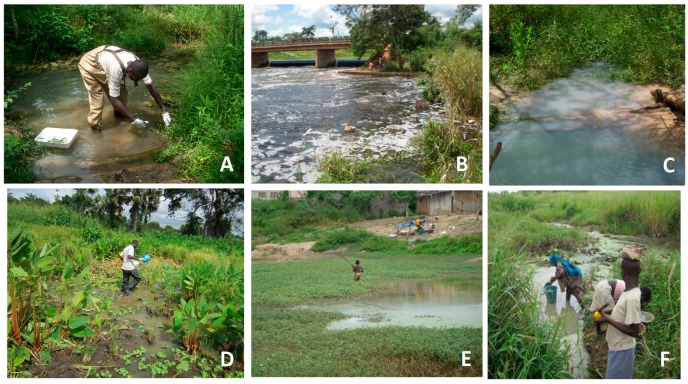
Some water bodies sampled within the study sites. (**A**) Plant sample collection in Tourou pond in Zaïbo, Daloa district; (**B**) La Lobo River in Zaïbo, Daloa district; (**C**) Nidrou1 pond in Gorodi, Daloa district; (**D**) sampling in Woudigné pond in Sokrogbo, Tiassalé district; (**E**) child fishing in Do pond in Léléblé, Tiassalé district; (**F**) inhabitants fetching water from Djapipo Barrage pond in Ahondo, Tiassalé district.

The environmental sample collection was performed during the dry season (17 to 26 September 2012). The water bodies sampled were selected based on frequency of use by community inhabitants for socio-economic activities as determined from questionnaire responses (Figure 2). Priority was given to water bodies that had undergone environment modifications based on activities such as farming, urbanization and construction of the hydroelectric dam.

Environmental sampling followed procedures described by Narh et al. [16]. Briefly, four different environmental matrices, soil, water filtrand, plant detritus and biofilms, were systematically collected using a quadrat-based sampling technique. Each sample was stored individually in 15- or 50-mL Falcon tubes with ethanol, kept on ice and transported to the laboratory for preservation at 4 °C until processing.

### 2.6. DNA Extraction, Conventional PCR and Sequencing Analysis

Total genomic DNA was extracted from lesion swabs and FNA samples using the QIAGEN DNeasy blood and tissue kit following the manufacturer’s protocol. DNA extraction from environmental samples was performed following the methods described previously [13,16]. All PCR reactions were performed in a A206 gradient thermal cycler (LonGene^®^) following described methods [16] using primers of the *Mycobacterium* 16S rRNA, the IS*2404* insertion sequence, the enoyl reductase (ER) domain of the mycolactone-producing gene, as well as variable number tandem repeat (VNTR) loci MIRU 1, ST1, Locus 6 and Locus 19 (Tables S1 and S2). Negative and positive controls were included for each run.

DNA extracts from environmental samples were initially screened for *Mycobacterium* spp. using the *Mycobacterium* 16S rRNA primer. Positive samples were then screened for mycobacteria harboring the IS*2404* insertion sequence using IS*2404* PCR. Samples that were IS*2404*-positive were screened for the presence of the ER domain of the mycolactone-producing gene. Screening of human samples followed a similar order, but without the initial 16S rRNA PCR.

In addition, 500-µL suspensions of each patient sample were decontaminated using the modified Petroff method [26]. The resulting pellet was streaked onto Lowenstein-Jensen slants and incubated for 36 weeks at 32 °C for isolation of mycobacteria.

All IS*2404*-positive samples were genotyped at four *M. ulcerans* VNTR loci; Locus 6, Locus 19, MIRU 1 and ST1. Length polymorphism was estimated from the size of the PCR product following separation on agarose gel and based on included positive controls as described by previous studies [13,16,19,27,28,29,30]. We used *M. ulcerans* 1615 strain, *M. liflandii* and *M. marinum* DL as the positive controls for the VNTR typing. Representative amplicons with good PCR products for each primer set from both human and environmental samples were sequenced using the forward and reverse primers in Sanger sequencing (Macrogen Inc., Netherlands). The sequences were queried against the NCBI nucleotide database using BLAST [31]. The E-value to consider homology and used for species ID confirmation was chosen to be below 10^−4^ or 0.0001. Search queries returning first hits with >70% nucleotide similarity were used to characterize isolates.

### 2.7. Statistical and Phylogenetic Analysis

Molecular data were recorded and stored in Microsoft Excel. The questionnaire data were analyzed using EpiData V 3.1. Alignments of queried sequences were performed using MAFFT [32], and the phylogenetic tree was drawn using FastTree [33] based on the maximum likelihood. FastTree incorporates the Jukes-Cantor model of nucleotide substitution; the distance is −0.75 × log(1 − 4/3 × d), where d is the proportion of positions that differ. When comparing two sequences, positions with gaps are ignored; when comparing two profiles, positions are weighted by their proportions of non-gaps. To account for the varying rates of evolution across sites, FastTree uses a single rate for each site (the ‘CAT’ (categories) approximation). To estimate the reliability of each split in the tree, FastTree computes local support values, based on 1000 resampling, with the Shimodaira–Hasegawa test. Statistical analysis was performed in Excel and Stata V12, and *p* < 0.05 for sample positivity comparison was considered statistically significant.

## 3. Results

### 3.1. Demographic Profile and Socio-Economic Activities Related to Water Bodies

**Figure 3:**
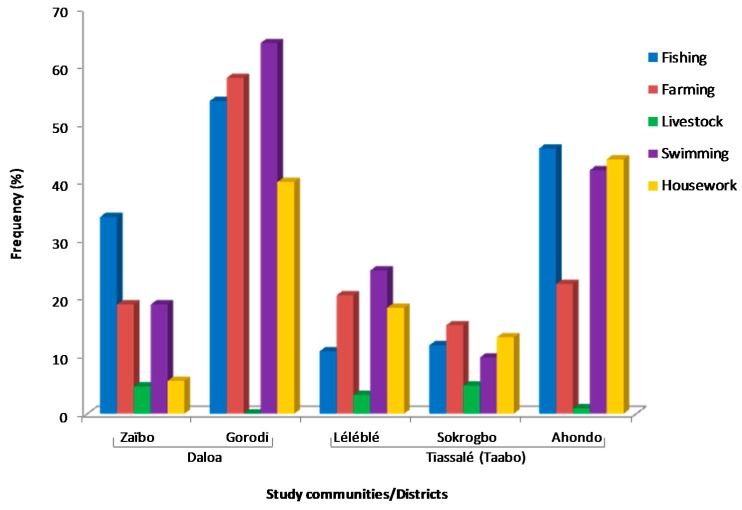
Proportion of respondents involved in water-related socio-economic activities. Farming included plant crop watering and irrigated crops; livestock represented livestock watering; swimming was considered as a children’s leisure activity; housework activities included water fetching for laundry, washing-up, crude food washing, drinking and bathing.

Of the 500 questionnaires, 58% were administered to males and 42% to females. Ages of respondents ranged from 18 to 98 years old with a mean of 42.5 years. However, the majority, 31% (95% CI, 27% to 35%) were within the 26 to 35 years age group. The proportions of water-related socio-economic activities identified by respondents within communities are shown in Figure 3.

The major socio-economic activities related to contact with environmental water bodies were fishing, farming (plant crop watering and irrigated crops), swimming and housework-related activities. Fishing was the main economic activity in the Ahondo and Zaïbo communities with 46% and 34% of respondents involved, respectively. In contrast, farming was the main economic activity in the Gorodi, Léléblé and Sokrogbo communities, with 58%, 20% and 15% of respondents involved, respectively. Swimming was also identified as the main water-related social activity for children, mostly in Gorodi (84%) and Ahondo (42%). Domestic activities including laundry, washing of dishes and raw farm produce and fetching water, were also undertaken by both children and adults, particularly in Ahondo (44%) and Gorodi (40%).

### 3.2. Confirmation of Clinical Cases and Incidence of BU within Communities

A total of 35 samples was collected from 30 BU suspected clinical cases (Table 1). Of these, 80% were collected from Tiassalé district (Taabo), whilst the remaining 20% were collected from Daloa district. Clinical data relating to BU suspected cases are shown in Table 1. The proportions of BU suspected cases were equal (50%) for males and females. The ages of patients ranged from 6 to 70 years with a mean age of 29 years. Based on the IS*2404* amplification results, 30% (95% CI; 14% to 46%) of the BU confirmed clinical cases were patients <18 years, while 70% (95% CI; 54% to 86%) were patients ≥18 years; this difference was statistically significant (*p* = 0.002). The majority of samples (94.3%) were taken from ulcers. The IS*2404* insertion sequence was detected in 33 samples out of 35 representing a BU confirmation positivity of 94% in the suspected clinical cases; that is, 28 out of 30 patients (three samples were patient duplicates). Thus, 100% of samples from Ahondo, Léléblé, Gorodi and Sokrogbo were IS*2404*-positive as compared to 33% from Zaïbo. Although ER PCR was attempted for all samples, only seven samples (21%) gave visible bands on agarose gel (Table 1, Figures S1 and S2).

Incubation on Löwestein-Jensen (LJ) culture slants for each patient for nine months showed that the majority of the tubes (*n* = 27) had no growth at the end of this period. We observed growth in a few tubes (*n* = 3), but upon further characterization using microscopy and PCR amplification using the same primers as above, none of the cultures were confirmed as *M. ulcerans*. 

### 3.3. Genotyping and Discrimination of Human Strains

VNTR-PCR genotyping at Locus 6, Locus 19, MIRU 1 and ST1 was attempted twice for all *IS2404*-positive samples, but PCR was successful for only seven (21%), based on at least two loci (Table 2). VNTR profiles were determined according to repeat number variation for PCR product sizes. Positive controls with known repeat numbers were run alongside isolates (Figure S3). Length polymorphisms were further confirmed with sequencing. However, for some samples, it was not possible to determine repeat numbers for VNTR loci, as a few sequence reads were not of good quality. Only reads with an average quality of 20 (Phred score) were further processed for repeat confirmation and phylogenetic analysis. Repeat sequences reported by Narh et al. [16] were used as references.

**Figure 4:**
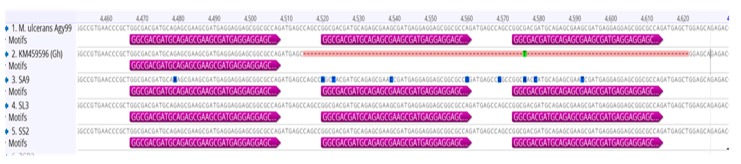
Sequence confirmation of MIRU 1 repeats in human samples. MIRU 1 reference sequences used were two orthologs from *M. ulcerans* Agy99 and the Ghanaian strain with Accession Numbers CP000325.1 and KM459596, respectively. The three human samples shown had a repeat variation of three for MIRU 1 as the reference strain *M. ulcerans* Agy99, while the Ghanaian strain had a repeat of one.

Figure 4 shows the MIRU 1 loci repeat variation for three human samples (SL3, SS2 and SA9), one Ghanaian *M. ulcerans* strain (GenBank ID, KM459596) and *M. ulcerans* Agy99. The human samples and *M. ulcerans* Agy99 each had a repeat of three. KM459596 (Ghana (Gh)) had one repeat as previously reported [16]. However, we detected a few single-nucleotide polymorphisms (SNPs) in the repeat sequences of some samples, including SA9.

VNTR profiles were arranged in order of MIRU 1, Locus 6, ST1 and Locus 19. A total of four allelic profiles, including (3, 1, 2, 2), (1, 1, _, _), (9, 1, _, _) and (_, 1, _, 4), were observed (Table 2). The two allelic profiles (3, _, 2, 2) and (3, 1, _, _) that were also observed could be other profiles or considered as incomplete profiles of (3, 1, 2, 2) (C profile). Unsuccessful amplification at a marker is indicated by a dashed line. The profile (1, 1, _, _) was similar to allelic repeats for MIRU 1 and Locus 6 in previously reported Ghanaian *M. ulcerans* genotypes, W, X and A [13,16]. For this study, Locus 6 and ST1 were monomorphic, with one and two repeats, respectively. In contrast, MIRU 1 gave repeat variations of 1, 3 and 9, and Locus 19 had two and four.

However, the presumptive profile (3, 1, 2, 2), associated with strains from Léléblé, Sokrogbo and Ahondo localities, was similar to profile C already reported in Ghana and Côte d’Ivoire. This length polymorphism was also present in the reference strain *M. ulcerans* Agy99 [13,34] (Figure 4). In Daloa district (Zaïbo and Gorodi communities), genotype profiles could not be determined due to unsuccessful amplification of the VNTR loci. 

### 3.4. Distribution of MPMs in Environmental Samples from Water Bodies

A total of 195 environmental samples was collected from 15 water bodies, 3 rivers and 12 ponds. These water bodies were identified as frequently used by inhabitants living within the study communities. At each water body, biofilms were collected in quintuplicates, water filtrands in duplicates, and both plant detritus and soils in triplicates. Each sample set collected was screened for mycobacterial 16S rRNA positivity. Those positive were then screened for the insertion sequence, IS*2404* (Table 3, Table 4 and Figure S4). We collected four matrices; 75 aquatic plant biofilms, 30 water filtrands, 45 plant detritus and 45 soil samples. Overall, the positivity rate for 16S rRNA was 53% (95% CI; 46% to 60%) while IS*2404* positivity (IS*2404* positives/16S rRNA positives) was 17% (95% CI; 9.3% to 24%). NTM positivity was highest in water filtrands (67%) and lowest in soils (44%) based on 16S rRNA detection. Interestingly, water filtrands and soil samples gave the highest (25%) and lowest (5%) positivity for MPMs, respectively based on IS*2404* DNA detection. All samples collected from Tourou pond in Zaïbo were positive for NTMs, but none from Djapipo barrage pond in Ahondo were positive. In a separate subgroup analysis, we observed that the mean NTM positivity for Daloa district, 71% (95% CI; 61% to 81%), was significantly (*p* <0.001) higher than Tiassalé district, 41% (95% CI; 32% to 50%). However, although the mean MPM positivity was also higher in Daloa, 18% (95% CI; 8% to 28.4%) than Tiassalé district, 17% (95% CI; 6% to 27%), the difference was not statistically significant (*p* = 0.820). VNTR-PCR for most IS*2404*-positive samples gave non-specific bands for all loci except for MIRU 1, which gave variable repeat sizes in 12% of the samples (Figure S5). Representatives of these were confirmed with sequencing.

### 3.5. Comparison of Human and Environmental MPM Strains

Based on read length and quality, IS*2404* sequences were used to identify and differentiate the MPM species in the human and environmental samples, as supplementary characterization (Table 5). The *IS2404* sequences identified all environmental samples, except SS1, SA2, SA10 and GGB4 (Gorodi Godo biofilm 4), as *M. liflandii*. One human sample, SA2, was identified as *M. ulcerans*. Interestingly, two human samples, SS1 and SA10, were identified as *M. pseudoshottsii* using the IS*2404* sequences.

### 3.6. Genotypes from Côte d’Ivoire Share Homology with Ghanaian Genotypes, but Maintain Unique Sub-Clusters

**Figure 5:**
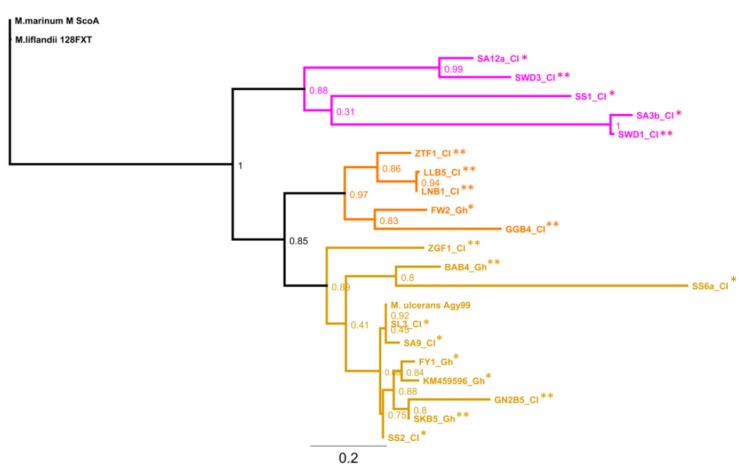
Phylogenetic tree of MPM isolates from human (*) and environmental (**) samples based on the MIRU 1 locus. Samples from the current study (Côte d’Ivoire) and Ghana are indicated with the suffix CI and Gh, respectively. Cluster 1 (CI 1) in purple and CI 2, in orange, are comprised of isolates mainly from Côte d’Ivoire. Cluster CI 3 shown in brown includes samples from Ghana and Côte d’Ivoire. The tree was rooted on *M. marinum*. The bootstrap (1000 resampling) values are shown beside the branches.

We sought to identify similarities between the genotypes identified in this study and those of previously-reported profiles in the sub-region. Figure 5 shows the phylogenetic relationship, based on the MIRU 1 locus, between genotypes from the current study (human and environmental) and those from Ghana, including *M. ulcerans* Agy99. KM459596_Gh, FW2_Gh, BAB4_Gh, FY1_Gh and SKB5_Gh are *M. ulcerans* strains reported by Narh et al. [16].

The tree was rooted using *M. marinum* as the common ancestor. Three main clusters (clades) were observed; clusters of samples from current study in Côte d’Ivoire (CI), CI 1 (purple), CI 2 (orange) and CI 3 (brown) with reliability (out of 1000 resampling) values of 88%, 97% and 89%, respectively. Five isolates, both human and environmental, from Côte d’Ivoire (purple) formed CI 1. The second cluster (CI 2) was comprised of four environmental isolates from Côte d’Ivoire and one human *M. ulcerans* isolate from Ghana. The third cluster (CI 3) was comprised of four environmental and seven human isolates from both Ghana and Côte d’Ivoire. This clade was comprised of smaller sub-clades.

## 4. Discussion

*Mycobacterium ulcerans* infection is prevalent in most rural communities in Côte d’Ivoire, but there is little information on the epidemiology of MPMs in the environment or whether they may be responsible for some of the human disease cases. It has become increasingly apparent that close contacts with contaminated aquatic ecosystems are major risk factors for *M. ulcerans* infection. Our previous findings in Ghana suggested that source tracking of contaminated water bodies is pertinent in uncovering the elusive mode of transmission of the disease [16]. Extending in this, we explored the distribution of MPMs in two BU endemic districts in Côte d’Ivoire and confirmed that common mycobacterial strains could be detected in humans and their proximal aquatic environments.

PCR detection of the IS*2404* and ER genes, supported with sequencing, was the confirmatory test for *M. ulcerans* infection in suspected cases. We were however unsuccessful in our attempts to isolate *M. ulcerans* from the LJ culture slants. Based on the sequence data, a lower temperature incubation other than 32 °C may have been helpful in the isolation and growth of the MPMs. This assertion was previously alluded to in a study that showed the diversity of PCR positivity in culture-positive and -negative patient samples in Ghana [35]. We were therefore limited to the reliance on PCR and sequencing for our data interpretation. This is not an unusual occurrence in *M. ulcerans* transmission and epidemiological studies [15,16,34,36], and newer genotyping methods of sample DNA have positively corroborated this observation.

Most (94%) of the suspected human cases we obtained for this study were positive for IS*2404*. We observed that the proportion of BU-confirmed cases in this study was significantly lower in children (<18 years) than adults (≥18 years), suggesting a difference in prevalence among the age categories. However, the 95% CI (14% to 46%) is comparable to prevalence estimates from WHO [37]. It is possible that other suspected cases might have been missed during our active case search and subsequently not reported to the health centers for treatment. Most children were at school during case searches. We also noted that, in most cases, infected individuals reported to health centers for treatment only when infection progressed to ulceration; only two out of 30 cases were nodules. Almost all communities showed 100% positivity in human samples, except Zaïbo community in Daloa district, with 33% positivity. Only 21% of samples showed ER positivity, with the majority in Ahondo. This slightly contrasts findings by Williamson and colleagues who successfully achieved amplification of the gene in a single reaction [15]. The failure to detect ER in other IS*2404*-positive samples could be attributed to low DNA concentration or lower bacterial abundance; some of the patients were on treatment during the study period. The use of more sensitive methods, including qPCR, if possible, could improve detection and, thus, should be recommended for laboratories in endemic countries [15,38]. In relation to the latter, the use of conventional PCR yields longer amplicons, which are useful for sequence analysis.

Our attempt to amplify four VNTR loci for IS*2404*-positive samples was challenging both for human and environmental samples. PCR protocols used here had been successfully used to genotype both human and environmental MPM strains collected in Ghana [16]. We suspected that our samples might have had low DNA concentrations as explained previously. The repeat variations of 1, 3 and 9 for MIRU 1 and 2 and 4 for Locus 19 suggest the presence of genetic diversity in MPM strains from Côte d’Ivoire. This length polymorphism in MIRU 1 also explained the limited, but shared homology with isolates from Ghana, as well as the overall genetic differentiation between the two countries. In Ghana, only one allele was reported for MIRU 1 and ST1 in contrast to two for both Locus 6 and Locus 19. In this study, three alleles were seen in MIRU 1 and two in Locus 19, for clinical isolates. Additionally, other MIRU 1 allelic sizes were also detected in environmental isolates. This suggests that MIRU 1 and Locus 19 may be more informative for typing isolates in Côte d’Ivoire. However, a larger sample size is needed to estimate the genetic diversity in these MPM populations. An ST1 monomorphic repeat of two is inconsistent with findings in Ghana where there was a polymorphism for the locus [16].

Interestingly, the allelic profile (3, 1, 2, 2) is similar to *M. ulcerans* C genotype, circulating in West Africa, particularly in Côte d’Ivoire [13,34]. Similar allelic profiles at some loci, e.g., (_, 1, 2, 2), suggest orthology to X (1, 1, 2, 2) and (1, 1, _, _) suggest orthology to W (1, 1, 2, 1) and A (1, 1, 1, 2) profiles carried by some Ghanaian isolates [16,35]. This may suggest similar strain lineages between the two countries. Interestingly, the Y profile (1, 2, 2, 1), which was found in both human and environmental samples in our previous study in Ghana [18], was not found in this study.

Our phylogenetic analysis showed that three major MPM populations might be circulating in Côte d’Ivoire. On one genetic background, there were isolates (Cluster CI 3) that shared sequence homology with both human and environmental *M. ulcerans* isolates from Ghana; notably, with two distinct Ghanaian strains, one with a single repeat of MIRU 1 sequence and the other with three repeats. The second MPM population (CI 2) consisted of only environmental isolates. These formed a clade with one human isolate, which was previously identified in Ghana as *M. ulcerans* [16]. The major branch connecting CI 2 and CI 3 suggests that both populations may have expanded from a common ancestor. The third cluster, CI 1, showed limited similarity to the Ghanaian strains, which suggests that they may be unique to Côte d’Ivoire. They were comprised of both human and environmental isolates. However, these findings should be interpreted cautiously since just a few sequences were used in the analysis. Although these observations were limited to diversity in one locus, our findings provide a hypothesis for using whole genome sequences to explore MPM patho-adaptions in both human and environmental reservoirs. 

The majority of the samples clustered with *M. ulcerans* sequences on the tree. Although IS*2404* sequencing identified *M. pseudoshottsii* DNA in the two human samples, separate analysis using the VNTR loci suggested they were more likely to be *M. ulcerans*. The discrepancies in MIRU 1 and IS*2404* identifications could be due to the presence of mixed genotypes. This is not uncommon, particularly in environmental samples [16]. IS*2404* is ubiquitous in most MPMs with high sequence similarity; thus, basing species identification, particularly environmental isolates, solely on the latter may lead to false conclusions. Thus, we suggest that IS*2404* typing of isolates, excluding clinical diagnosis, should be complemented with a full set of polymorphic markers. The samples with *M. pseudoshottsii* could be (1) a true *M. pseudoshottsii* infection or (2) an *M. ulcerans* with similar IS*2404* sequence to that of *M. pseudoshottsii*. These two hypotheses need further investigation with SNP typing and/or whole genome sequencing. The observance of *M. liflandii* in both environmental and human samples was not surprising. Comparative genome analysis and VNTR typing suggested that *M. liflandii* was an ecovar of *M. ulcerans* [16,39,40]. The allelic profile (3, _, 2, 2) observed here is similar to those reported for *M. liflandii* strains Xt128 and 1138 [13,35]. These observations are consistent with the phylogenetic clustering with *M. ulcerans*.

Transmission of MPMs could occur when individuals at risk come into contact with contaminated environments. Several overlaps occur in water bodies, through water-related activities [41]. In our study communities, a large proportion of respondents were engaged in agricultural activities, particularly fishing and farming. Children helped their parents on farms, and many bathed in the water bodies. These suggest frequent contact with water bodies, which are also sources of drinking water for most inhabitants. Although discrimination of MPMs from environmental samples was not as expected, their presence in nearly all water bodies based on IS*2404* amplification, suggests that they could be reservoirs and sources of infection. MPMs were detected in nearly all water bodies, except one. 

The two main clusters on the phylogenetic tree comprise both human and environmental isolates (Figure 5). This neighbor joining suggests strain relatedness between the human and environmental isolates. For instance, the branch between the environmental sample SWD1_CI and the human sample SS1_CI suggested a monophyletic relationship. Both samples were from the same community (Sokrogbo, Tiassalé district), which suggests that the water bodies there could be sources of infection. Environmental contaminants could inhibit PCR reactions; we observed evidence of many human activities in some water bodies, as some were cloudy and had sediments when filtered on the Whatman filters.

Overall, we observed a significantly higher positivity of NTMs than MPMs in the environment. Thus, there may be a broader and persistent distribution of many *Mycobacterium* spp. in endemic communities. However, MPM presence may be in select foci, which is consistent with the geographic epidemiology of the disease [42]. MPM presence was not significantly higher in Daloa than in Tiassalé. We observed higher 16S and IS*2404* positivity in water filtrands. This contrasts with reports of higher positivity in biofilm samples from Ghana [13,16]. Standardization of the sampling protocols between this study and previous studies in Ghana suggests that this discrepancy could be due to the water bodies sampled. In this study, 80% of the water bodies were ponds compared to the equal number of ponds, streams and rivers in the Ghanaian study [16]. However, the association between 16S and IS*2404* positivity is consistent with previous data suggesting that the presence of *Mycobacterium* spp. may not necessarily indicate the presence of some MPMs, particularly *M. ulcerans* [16]. Although the environmental and human strains shared IS*2404* sequence similarity, the absence of VNTR profiles on the environmental strains provides little evidence to track infections in this study.

Finally, we recommend the standardization of protocols employed in BU research; particularly, those employed in routine environmental sampling and PCR analysis [13,16,35].

## 5. Conclusions

We successfully demonstrated that most of the water bodies in this study were contaminated with MPMs, notably *M. ulcerans*, which could infect inhabitants involved in water-related socio-economic activities. The shared length polymorphisms at the VNTR loci in our strains and other studies in both Côte d’Ivoire and Ghana suggest common ancestral lineages. The allelic copy number in MIRU 1 and Locus 19 suggests diversity in MPM strains from Côte d’Ivoire.

Using standardized sampling and genotyping protocols from Ghana allowed the comparison of datasets. The latter could help explain the role of different ecologies and demography in BU. This study also suggests that distribution, but not the type of MPMs in two BU endemic districts may be significantly different.

The tentative detection of other MPMs like *M. pseudoshottsii* in human infections highlights their characteristic as opportunistic pathogens. Whole genome analysis can provide insights into possible niche adaptations in the human host. These co-infections may have implications for resistance to drugs used in BU treatment, which only focus on *M. ulcerans* strains, and lead to larger policy implications in treatment strategies.

## Figures and Tables

**Table 1:** Clinical data on Buruli ulcer (BU) suspected cases within communities. ER, enoyl reductase.

Clinical History							PCR Test	
District	Community	Sample Code	Sex	Age (years)	Lesion	Specimen	IS*2404*	ER
Daloa	Zaïbo	SZ1	M	27	ulcer	swab	Pos	Neg
		SZ2	F	65	ulcer	swab	Neg	Neg
		SZ3	F	27	ulcer	swab	Neg	Neg
	Gorodi	FG1	M	21	nodule	FNA	Pos	Neg
		SG2	F	29	ulcer	swab	Pos	Neg
		SG3	F	70	ulcer	swab	Pos	Neg
		SG4	F	48	ulcer	swab	Pos	Neg
Tiassalé (Taabo)	Léléblé	SL1	M	7	ulcer	swab	Pos	Neg
		SL2	M	7	ulcer	swab	Pos	Neg
		SL3	F	43	ulcer	swab	Pos	Pos
		SL4a	M	10	ulcer	swab	Pos	Neg
		SL4b	M	10	ulcer	swab	Pos	Neg
	Sokrogbo	SS1	M	60	ulcer	swab	Pos	Neg
		SS2	F	10	ulcer	swab	Pos	Pos
		SS3	F	56	ulcer	swab	Pos	Neg
		SS4	F	19	ulcer	swab	Pos	Neg
		SS5	F	30	ulcer	swab	Pos	Neg
		SS6a	M	17	ulcer	swab	Pos	Neg
		SS6b	M	17	ulcer	swab	Pos	Neg
		SS7	F	20	ulcer	swab	Pos	Neg
		SS8	F	30	ulcer	swab	Pos	Neg
		SS9	M	36	ulcer	swab	Pos	Neg
	Ahondo	SA1	M	12	ulcer	swab	Pos	Pos
		SA2	M	50	ulcer	swab	Pos	Neg
		SA3a	M	25	ulcer	swab	Pos	Neg
		SA3b	M	25	ulcer	swab	Pos	Neg
		FA4	M	49	nodule	FNA	Pos	Neg
		SA6	F	13	ulcer	swab	Pos	Pos
		SA7	F	18	ulcer	swab	Pos	Neg
		SA8	M	44	ulcer	swab	Pos	Neg
		SA9	F	6	ulcer	swab	Pos	Pos
		SA10	M	12	ulcer	swab	Pos	Pos
		SA11	M	10	ulcer	swab	Pos	Pos
		SA12a	M	44	ulcer	swab	Pos	Neg
		SA12b	M	44	ulcer	swab	Pos	Neg

M: male; F: female; FNA: fine needle aspiration; SZ: swab lesion Zaïbo; FG: FNA Gorodi; SG: swab lesion Gorodi; SL: swab lesion Léléblé; SS: swab lesion Sokrogbo; SA: swab lesion Ahondo; FA: FNA Ahondo; Pos: positive; Neg: negative. IS*2404* positivity: Zaïbo (33%), Gorodi (100%), Léléblé (100%), Sokrogbo (100%) and Ahondo (100%).

**Table 2:** VNTR typing and allelic profile from BU confirmed clinical samples and African mycolactone-producing mycobacteria (MPM) genotypes from published data.

**BU Confirmed Clinical Cases**							
**Community**	**Sample Code**	**Allelic Profiles**				**Genotype**	**Reference**
		**MIRU1**	**Locus 6**	**ST1**	**Locus 19**		
Léléblé	SL2	1	1	N/D	N/D	undetermined	current study
	SL3	3	N/D	2	2	C-	[13,34]
Sokrogbo	SS1	9	1	N/D	N/D	undetermined	current study
	SS2	3	N/D	2	2	C-	[13,34]
Ahondo	SA2	N/D	1	N/D	4	undetermined	current study
	SA9	3	1	N/D	N/D	C-	[13,34]
	SA10	3	1	2	2	C	[13,34]
**Published African MPM Genotypes**							
**Allelic Profiles**						**Genotype**	**Reference**
**MPM Strain**		**MIRU1**	**Locus 6**	**ST1**	**Locus 19**		
*M. ulcerans*		1	1	1	2	A	[35]
		3	1	1	2	B	
		3	1	2	2	C	
		1	1	2	2	D	
Mycolactone-producing *M. marinum* and *M. pseudoshottsii*		1	4	2	2	MPM	
Mycolactone-producing *M. liflandii*		1	2	2	1	MPML	

N/D: non-amplified locus; SL: swab lesion Léléblé; SS: swab lesion Sokrogbo; SA: swab lesion Ahondo; C-: incomplete profile of genotype C.

**Table 3:** 16S rRNA and IS*2404* positivity in water bodies.

	Communities	Water Bodies	16S rRNA N° (%)	IS*2404* N° (%)
Daloa	Zaïbo	Gbouwa pond	9/13 (69.23%)	4/9 (44.44%)
		Tourou pond	13/13 (100%)	1/13 (7.69%)
		La Lobo River	11/13 (84.62%)	2/11 (18.18%)
	Gorodi	Godo River	7/13 (53.85%)	1/7 (14.26%)
		Nidrou 1 pond	5/13 (38.46%)	0/5 (0%)
		Nidrou 2 pond	10/13 (76.92%)	2/10 (20%)
	Sub-total		55/78 (71.0%)	10/55 (18.2%)
Tiassalé	Léléblé	N’ziba pond	11/13 (84.62%)	1/11 (9.09%)
		Do pond	7/13 (53.85%)	0/7 (0%)
		Barrage pond	5/13 (38.46%)	1/5 (20%)
		Lahôbloua pond	10/13 (76.92%)	1/10 (10%)
	Sokrogbo	Woudigné pond	4/13 (30.77%)	2/4 (50%)
		Barrage 1 pond	4/13 (30.77%)	0/4 (0%)
		Barrage 2 pond	2/13 (15.38%)	0/2 (0%)
	Ahondo	Djapipo Barrage pond	0/13 (0%)	0/0 (0%)
		Bandama River	5/13 (38.46%)	2/5 (40%)
	sub-total		48/117 (41%)	7/48 (14.5%)
	Overall Prevalence		103/195 (52.82%)	17/103 (16.50%)

**Table 4:** 16S rRNA and IS*2404* positivity in environmental matrices.

	Sample Matrices	16S rRNA N° (%)	IS*2404* N° (%)
Sample Type	Plant biofilm	38/75 (50.67 %)	7/38 (18.42%)
	Water filtrand	20/30 (66.67 %)	5/20 (25%)
	Plant detritus	25/45 (55.56 %)	4/25 (16%)
	Soil	20/45 (44.44 %)	1/20 (5%)
	Total	103/195 (52.82 %)	17/103 (16.50%)

**Table 5:** Identification of MPM strains based on IS*2404* sequence similarity.

**Clinical Isolates**				
**District**	**Community**		**Type of Sample (ID)**	***IS2404* Identity**
Tiassalé (Taabo)	Léléblé		Lesion swab (SL3)	98% for *M. liflandii* 128 FXT
	Sokrogbo		Lesion swab (SS1)	97% for *M. pseudoshottsii* L15
			Lesion swab (SS2)	97% for *M. liflandii* 128 FXT
			Lesion swab (SS6a)	N/D
	Ahondo		Lesion swab (SA2)	96% for *M. ulcerans* Agy99
			Lesion swab (SA3b)	N/D
			Lesion swab (SA9)	N/D
			Lesion swab (SA10)	95% for *M. pseudoshottsii* L15
			Lesion swab (SA12a)	N/D
**Environmental Isolates**				
Daloa		Zaïbo	Gbouwa pond/water filtrand (ZGF1)	N/D
			Tourou pond/water filtrand (ZTF1)	89% for *M. liflandii* 128 FXT
		Gorodi	Godo River/plant biofilm (GGB4)	96% for *Mycobacterium* sp. YM-1
			Nidrou2 pond/plant biofilm (GN2B5)	97% for *M. liflandii* 128 FXT
Tiassalé (Taabo)		Léléblé	N’Ziba pond/plant biofilm (LNB1)	99% for *M. liflandii* 128 FXT
			Lahôbloua pond/plant biofilm (LLB5)	N/D
		Sokrogbo	Woudigné pond/plant detritus (SWD1)	N/D
			Woudigné pond/plant detritus (SWD3)	99% for *M. liflandii* 128 FXT

N/D, Not identified. Percentage (%) identity of the IS*2404* sequence to reference orthologs.

## Supplementary Materials

The following are available online at http://www.mdpi.com/2414-6366/2/1/3/s1. **Figure S1.** Amplification of IS2404 from some clinical samples. M: 100 bp DNA ladder; Ctl+: positive control (*M. ulcerans* strain); Ctl-: negative control (sterile water); SA10-SA12b, SS3, SS4, SS8, SS9, SA3b-SG4: clinical samples tested. **Figure S2.** Amplification of enoyl reductase (ER) gene from clinical samples. M: 100 bp DNA ladder; Ctl+: positive control (*M. ulcerans* strain); Ctl-: negative control (sterile water); SS1-FA4, SA6-SG4, SA9-SA12b: clinical samples tested. **Figure S3.** Amplification of VNTR loci from clinical samples. A: Locus 6 amplification; B: ST1 amplification. M: 100 bp DNA ladder; Ctl+: positive control (*M. Marinum* DL strain); Ctl-: negative control (sterile water); SS1-SG4: clinical samples tested. **Figure S4.** Amplification of IS2404 from positive 16S rRNA environmental samples. M: 100 bp DNA ladder; Ctl+: positive control (*M. Marinum* strain); Ctl-: negative controls (sterile water); ZLB1-LNS1: environmental samples tested. **Figure S5**. Amplification of VNTR loci from environmental samples. A: MIRU 1 amplification; B: Locus 6 amplification. M: 100 bp DNA ladder; Ctl+ 1: positive control (*M. Marinum* DL strain); Ctl+ 2: positive control (*M. ulcerans* strain); Ctl-: negative control (sterile water); ZGF1-ABS2 (A), ZGB31-ZLF2 (B): environmental samples tested. **Table S1.** Primers used for identification of MPMs. **Table S2.** Size of PCR product of VNTR loci and associated repeat number.
