# Supplementary material for: Distribution and Risk of Mycolactone-Producing Mycobacteria Transmission within Buruli Ulcer Endemic Communities in Côte d’Ivoire
Source: Trop Med Infect Dis. 2017 Feb 26;2(1):3. doi: 10.3390/tropicalmed2010003 (PMC6082052; doi:10.3390/tropicalmed2010003)
Supplement: Supplementary file 1 [file tropicalmed-02-00003-s001.pdf]

Distribution and Risk of Mycolactone-Producing Mycobacteria Transmission within Buruli Ulcer Endemic Communities in Côte d'Ivoire

Christelle Dassi, Lydia Mosi, Charles A Narh, Charles Quaye, Danièle O. Konan, Joseph A. Djaman and Bassirou Bonfoh

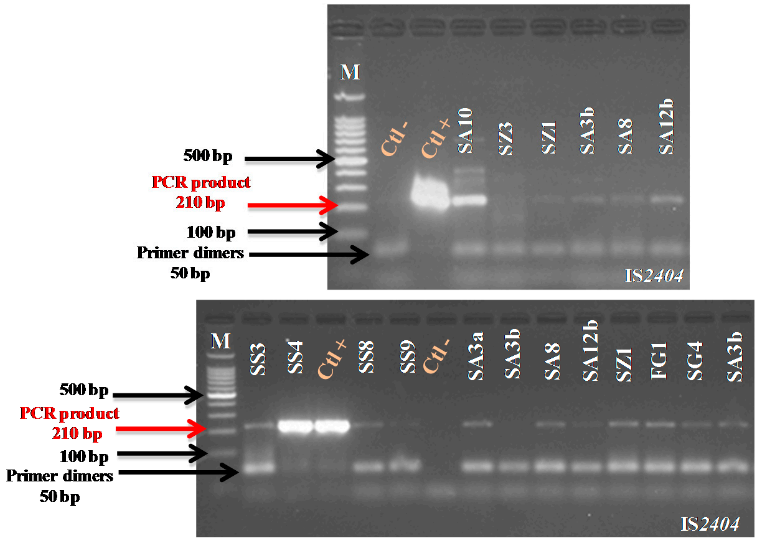

Figure S1. Amplification of IS*2404* from some clinical samples. M: 100 bp DNA ladder; Ctl+: positive control (*M. ulcerans* strain); Ctl-: negative control (sterile water); SA10-SA12b, SS3, SS4, SS8, SS9, SA3b-SG4: clinical samples tested.

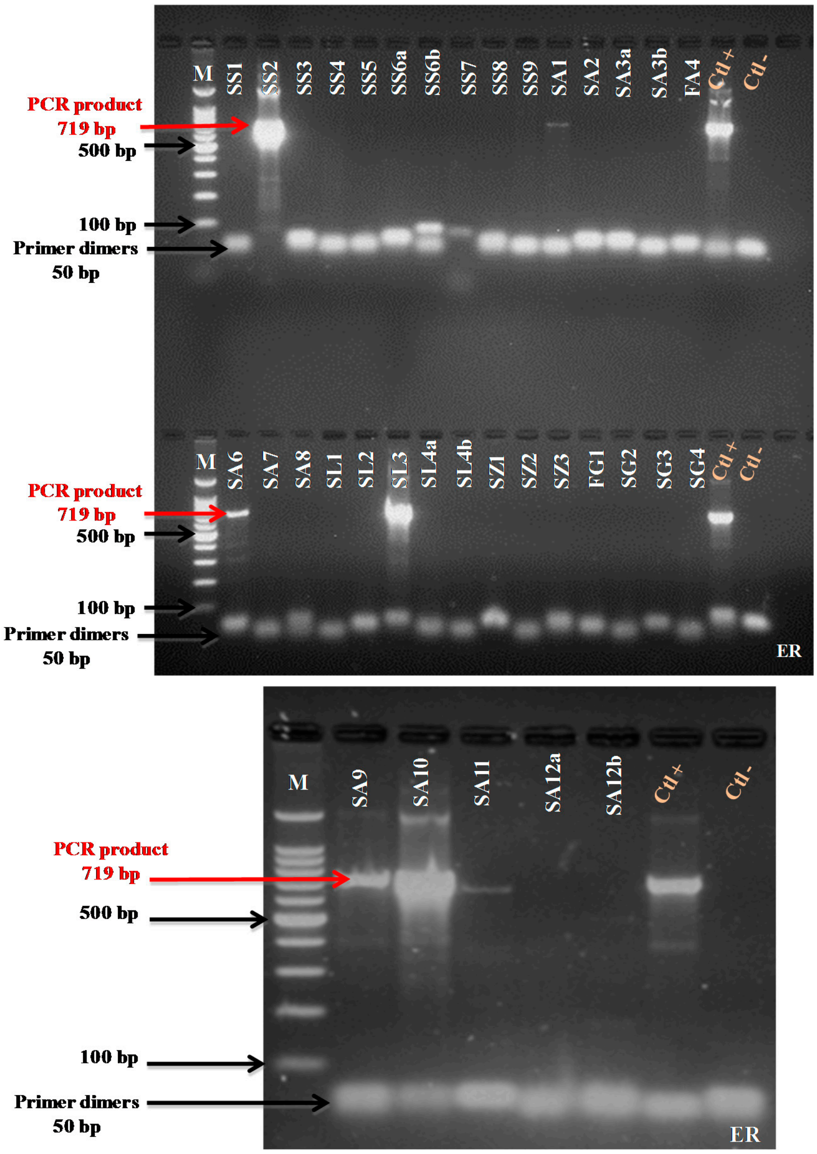

Figure S2. Amplification of enoyl reductase (ER) gene from clinical samples. M: 100 bp DNA ladder; Ctl+: positive control (*M. ulcerans* strain); Ctl-: negative control (sterile water); SS1-FA4, SA6-SG4, SA9-SA12b: clinical samples tested.

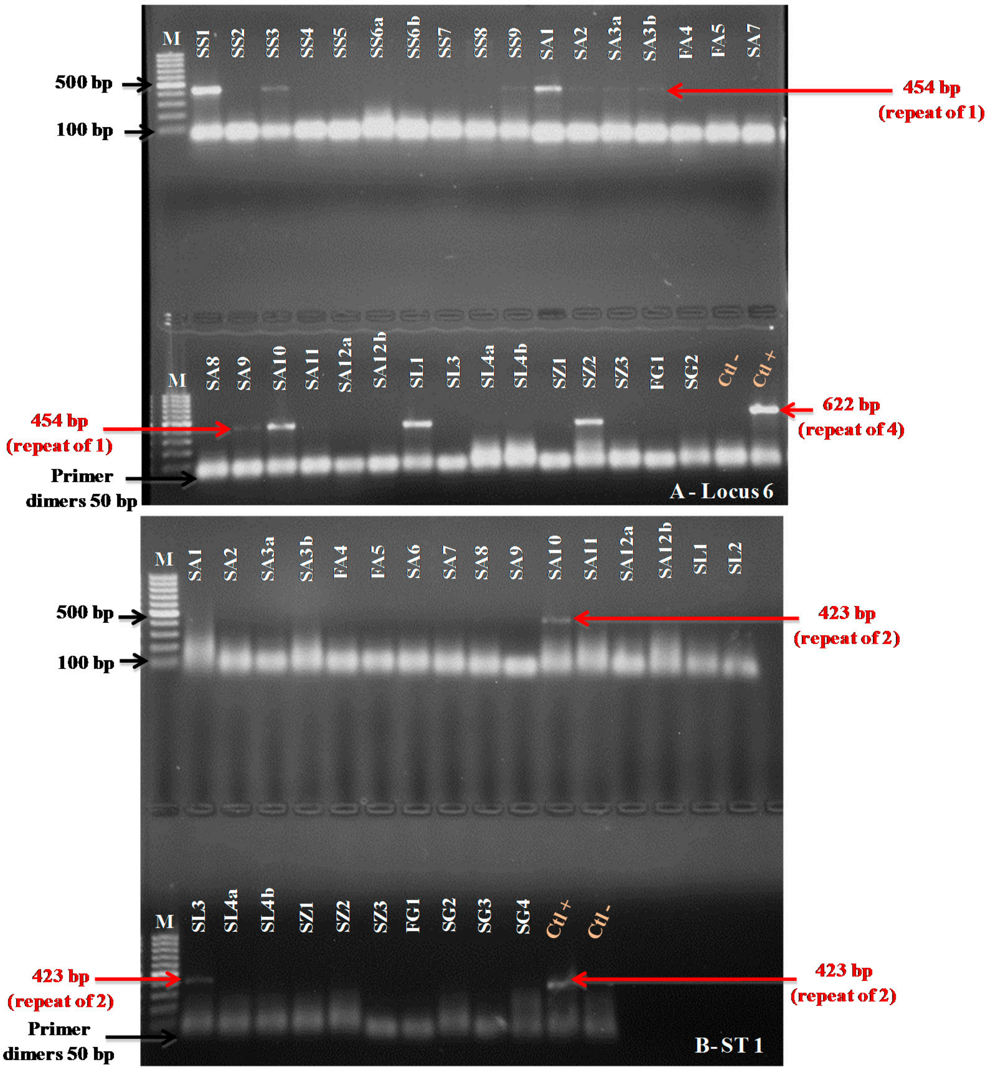

Figure S3. Amplification of VNTR loci from clinical samples. A: Locus 6 amplification; B: ST1 amplification. M: 100 bp DNA ladder; Ctl+: positive control (*M. Marinum* DL strain); Ctl-: negative control (sterile water); SS1-SG4: clinical samples tested.

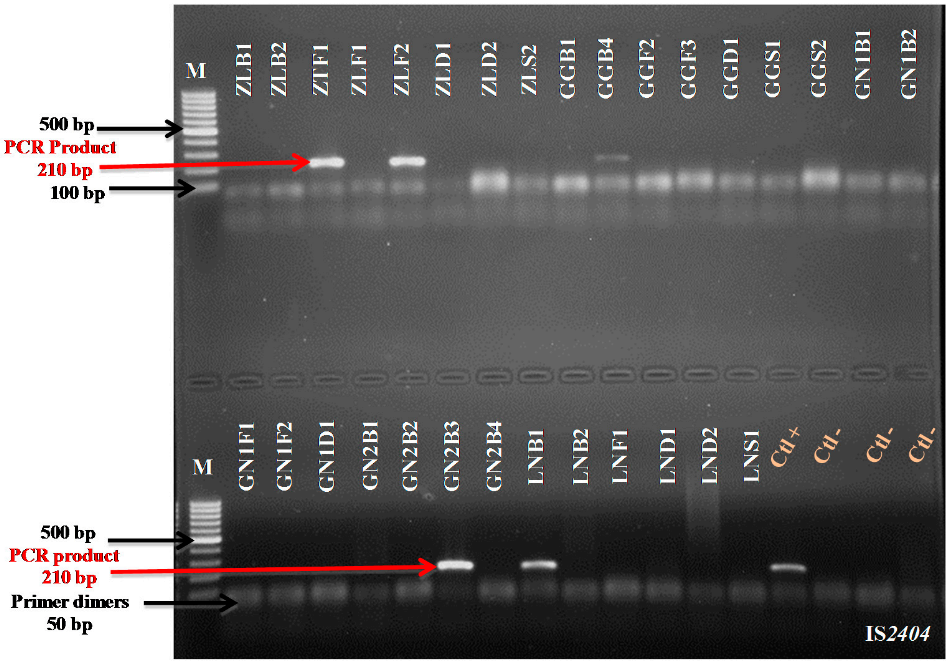

Figure S4. Amplification of IS*2404* from positive 16S rRNA environmental samples. M: 100 bp DNA ladder; Ctl+: positive control (*M. Marinum* strain); Ctl-: negative controls (sterile water); ZLB1-LNS1: environmental samples tested.

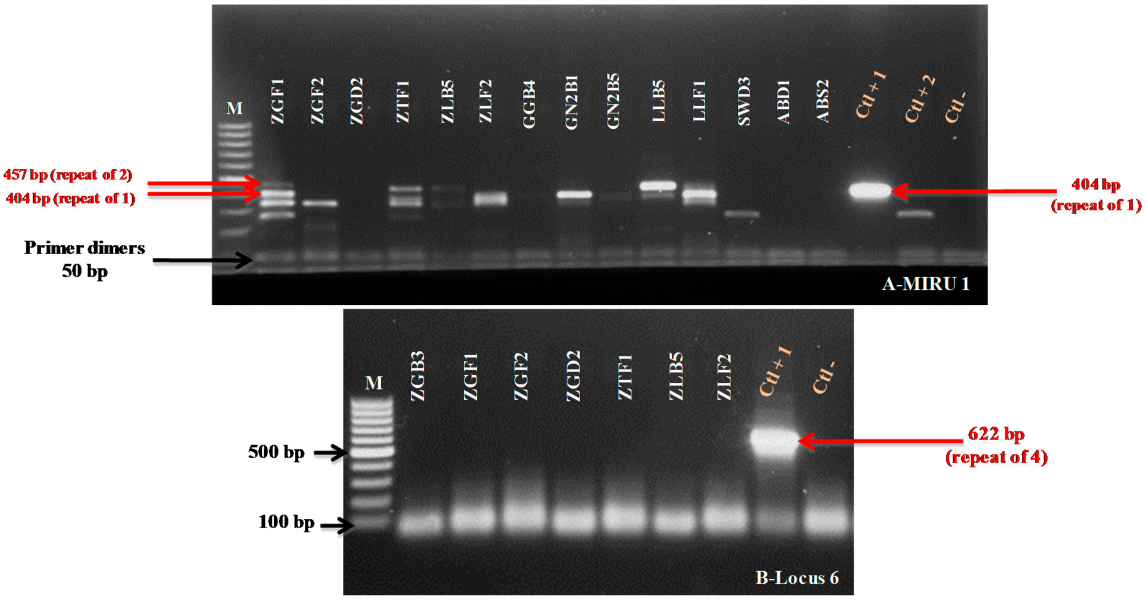

Figure S5. Amplification of VNTR loci from environmental samples. A: MIRU 1 amplification; B: Locus 6 amplification. M: 100 bp DNA ladder; Ctl+ 1: positive control (*M. Marinum* DL strain); Ctl+ 2: positive control (*M. ulcerans* strain); Ctl-: negative control (sterile water); ZGF1-ABS2 (A), ZGB31-ZLF2 (B): environmental samples tested.

Table S1. Primers used for identification of MPMs.

Primer Name	Forward and Reverse Primer Sequences	PCR Product Size	Reference
16S rRNA	PA:5'-AGAGTTTGATCCTGGCTCAG-3'<br>MSHA: 5′-AAAAAGCGACAAACCTACGAG-3'	620 bp	[1]
IS*2404* (nested 1)	pGp1:5′-AGGGCAGCGCGGTGATACGG-3′<br>pGp2: 5′-CAGTGGATTGGTGCCGATCGAG-3′	400 bp	[2]
IS*2404* (nested 2)	pGp3: 5′-GGCGCAGATCAACTTCGCGGT-3′<br>pGp4: 5′-CTGCGTGGTGCTTTACGCGC-3	210 bp	[2]
ER	F:5′-GAGATCGGTCCCGACGTCTAC-3′<br>R:5′-GGCTTGACTCATGTCACGTAAG-3′	719 bp	[3]
Locus 6	F-5′-GACCGTCATGTCGTTCGATCCTAGT-3′<br>R-5′-GACATCGAAGAGGTGTGCCGTCT-3′	variable	[3]
Locus 19	F-5′-CCGACGGATGAATCTGTAGGT-3′<br>R-5′-TGGCGACGATCGAGTCTC-3′	variable	[3]
MIRU 1	F-5′-GCTGGTTCATGCGTGGAAG-3′<br>R-5′-GCCCTCGGGAATGTGGTT-3′	variable	[3]
ST1	F-5′-CTGAGGGGATTTCACGACCAG-3′<br>R-5'-CGCCACCCGCGGACACAGTCG-3'	variable	[3]

Table S2. Size of PCR product of VNTR loci and associated repeat number.

VNTR Loci	Repeat Lenght (bp)	PCR Product Size in bp (Associated Repeat Number)								
MIRU 1	53	404 (1)	457 (2)	510 (3)	563 (4)	616 (5)	669 (6)	722 (7)	775 (8)	828 (9)
Locus 6	56	454 (1)	510 (2)	566 (3)	622 (4)	678 (5)	734 (6)	790 (7)	846 (8)	902 (9)
ST1	54	369 (1)	423 (2)	477 (3)	531 (4)	585 (5)	639 (6)	693(7)	747 (8)	801 (9)
Locus 19	56	288 (1)	344 (2)	400 (3)	456 (4)	512 (5)	568 (6)	624 (7)	680 (8)	736 (9)

References

1. Hughes, M.S.; Skuce, R.A.; Beck, L.A.; Neill, S.D. Identification of mycobacteria from animals by restriction enzyme analysis and direct DNA cycle sequencing of polymerase chain reaction-amplified 16S rRNA gene sequences. *J. Clin. Microbiol.* **1993**, *31*, 3216–3222.
2. Ablordey, A.; Amissah, D.A.; Aboagye, I.F.; Hatano, B.; Yamazaki, T.; Sata, T.; Ishikawa, K.; Katano, H. Detection of *Mycobacterium ulcerans* by the loop-mediated isothermal amplification method. *PLoS Negl. Trop. Dis.* **2012**, *6*, e1590.
3. Williamson, H.R.; Benbow, M.E.; Nguyen, K.D.; Beachboard, D.C.; Kimbirauskas, R.K.; McIntosh, M.D.; Quaye, C.; Ampadu, E.O.; Boakye, D.; Merritt, R.W.; et al. Distribution of *Mycobacterium ulcerans* in Buruli ulcer endemic and non-endemic aquatic sites in Ghana. *PLoS Negl. Trop. Dis.* **2008**, *2*, e205.
